# Identification of colloidal haze protein in Chinese rice wine (Shaoxing Huangjiu) mainly by matrix‐assisted laser ionization time‐of‐flight mass spectrometry

**DOI:** 10.1002/fsn3.1655

**Published:** 2020-06-23

**Authors:** Guangfa Xie, Jiongping Han, Xueyuan Han, Qi Peng, Jianwei Fu, Chi Shen, Jianqiu Sun, Junyong Sun, Jian Lu, Yin Lu, Guolong Li

**Affiliations:** ^1^ College of Biology and Environmental Engineering Zhejiang Shuren University Shaoxing China; ^2^ School of Medicine Shaoxing University Shaoxing China; ^3^ School of Life Sciences Shaoxing University Shaoxing China; ^4^ National Engineering Research Center for Chinese Huangjiu Shaoxing China; ^5^ National Engineering Laboratory for Cereal Fermentation Technology Jiangnan University Wuxi China; ^6^ Shaoxing Jianhu Brewing Co., Ltd Shaoxing China

**Keywords:** amino acid, Chinese rice wine, colloidal haze, haze protein, Huangjiu

## Abstract

As one of the three most famous brewed wines in the world, Chinese rice wine is made from rice and husked millet, containing 14 percent to 20 percent alcohol. Highly original, yellow wine brewing techniques are regarded as the model of the wine brewing industry in Asia. Shaoxing Huangjiu is produced in Zhejiang province and remains the oldest and most representative Chinese rice wine. During storage, Shaoxing Huangjiu is susceptible to environmental disturbance and produces colloidal haze to result in turbidity. In this study, the main composition and source of colloidal haze protein in Shaoxing Huangjiu were analyzed by two‐dimensional electrophoresis and matrix‐assisted laser ionization time‐of‐flight tandem mass spectrometry (MALDI‐TOF/TOF MS). The results showed that the proteins in colloidal haze mainly consisted of oat protein b1, oat‐like protein, di‐amylase inhibitor, pathogenesis‐related protein, pathogenesis‐related protein‐4, chitinase II derived from wheat and oat‐like protein, and beta‐amylase derived from rice. The amino acid composition and secondary structure of haze protein and supernatant protein in Huangjiu were further explored by high‐performance liquid chromatography and Fourier transform infrared spectroscopy. The study has broadened knowledge of the main composition and source of colloidal haze protein in Shaoxing Huangjiu. The corresponding results indicated that the amino acid composition from colloidal haze had the main characteristics of high hydrophobicity and low water solubility.

## Graphical abstract

In this study, the main composition and source of colloidal haze protein in Chinese rice wine (Shaoxing Huangjiu) were analyzed by two‐dimensional electrophoresis and matrix‐assisted laser ionization time‐of‐flight tandem mass spectrometry (MALDI‐TOF/TOF MS). Meanwhile, the amino acid composition and secondary structure of haze protein and supernatant protein in Huangjiu were further explored by high‐performance liquid chromatography and Fourier transform infrared spectroscopy.

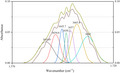

## 1 INTRODUCTION

Huangjiu (Chinese fermented rice wine), a popular and traditional alcoholic beverage in China, is a kind of colloid solution with complex composition and rich nutrition. Generally, Huangjiu is produced from rice with “wheat Qu” and yeast by simultaneous saccharification and fermentation, following filtration, clarification, and sterilization. During Huangjiu brewing, the degradation of raw materials by co‐fermentation with yeast, lactic acid bacteria, and fungi contributes to the production of abundant proteins, amino acids, vitamins, mineral elements, and oligosaccharides (Lv et al., 2016). Thus, Huangjiu is honored as “liquid cake” for its rich in nutrient substances (Yang et al., 2019). However, since Huangjiu could be affected by light, vibration, and oxygen during storage, its colloidal balance is broken to further result in the loss of light, flocculation, and colloidal haze (Van Sluyter et al., 2015).

In recent years, many researchers have become concerned about the colloidal haze components of Huangjiu (Lin, BAI, & ZOU, 2005; Xie, Meng, & Zhou, 2002). The study on the colloidal haze components in altar and bottled Huangjiu showed that the crude protein content of colloidal haze in the altar was 34.66% and that in the bottled Huangjiu accounted for 50.56% (Yang, Zeng, Chen, & Xiao, 2003). Similarly, our previous study revealed that the crude protein content of colloidal haze reached 50.60% in bottled Huangjiu, and further, the large molecular protein accounted for 72.62% of the total protein content (Xie, Meng, et al., 2002). According to the above studies, it was shown that protein was the main component of colloidal haze of Huangjiu. However, the increasing large proportion of large molecular weight proteins in colloidal haze did not decrease the stability of Huangjiu (Jiao, Xu, & Jin, 2017; Lin & BAI, 2005; YANG, YU, & WEI, 2005). This was speculated to relate to certain types of proteins in Huangjiu that were prone to colloidal haze.

In order to understand which types of proteins contribute most significantly to haze, two‐dimensional electrophoresis (2‐DE) and matrix‐assisted laser desorption ionization time‐of‐flight tandem mass spectrometry (MALDI‐TOF/TOF MS) were used to trace the types and sources of colloidal haze in Shaoxing Huangjiu. The amino acid composition and secondary structure of the colloidal haze in Shaoxing Huangjiu were further analyzed by high‐performance liquid chromatography (HPLC) and Fourier transform infrared spectroscopy (FTIR), respectively. This research is to help understand the colloidal haze of Shaoxing Huangjiu and provide new theoretical guidance for solving the problem of colloidal haze.

## 2 MATERIALS AND METHODS

### 2.1 Materials and instruments

Huangjiu samples of different ages with alcohol content ≥15.5% vol and total sugar 15.1–40.0 g/L were from Zhejiang Guyuelongshan Shaoxing Wine Co., Ltd., Tapai Shaoxing Wine Co., Ltd., and Kuaijishan Shaoxing Wine Co., Ltd.; protein electrophoresis system (mini‐protein 3 cell), Bio‐Rad Laboratories Co., Ltd.; high‐speed freezing centrifuge, Sigma Co., Ltd.; UltrafleXtreme series time‐of‐flight mass spectrometer, Bruker Co., Ltd.; UV‐2100 UV‐VIS spectrophotometer, Yunick Shanghai Instruments Co., Ltd.; WGZ‐2‐PJ turbidimeter, Shanghai Xinrui instrument Co., Ltd.; K2300 Kjeldahl Nitrometer, Foss Analytical Co., Ltd.; amino acid high‐performance liquid chromatograph, Agilent Technologies Co., Ltd., and Fourier transform infrared spectrometer, Nexus Nicolet 470, Thermo Nicolet Co., Ltd.

### 2.2 Experimental methods

#### 2.2.1 Preparation of haze protein from Huangjiu

The wine sample of 500 ml was stored at room temperature for a few days until cloudy precipitation occurred. Then, the sample was centrifuged (12,000 g, 30 min) to collect sediment, suspended in ultra‐pure water, and the above process was repeated once. The clean sediment was dissolved in 2% aqueous ammonia, salted out with 80% saturated ammonium sulfate, then desalted in 1,000 Da dialysis bag at 4°C for 48 hr, and lyophilized to obtain haze protein samples (Pocock, Alexander, Hayasaka, Jones, & Waters, 2007).

#### 2.2.2 Two‐dimensional electrophoresis

SDS‐PAGE was performed in 12.5% polyacrylamide gels using a Bio‐Rad Mini‐Protean III cell equipment. For 2‐DE, protein samples were applied by overnight rehydration into immobilized pH gradient (IPG) strips of pH 3–10 ([General Electric Company] GE Healthcare Life Sciences China), subjected to isoelectric focusing electrophoresis (IEF), and then resolved in 12.5% polyacrylamide gels. Following electrophoresis, protein gels were visualized with comas bright blue R 250.

#### 2.2.3 Haze protein identification by MALDI‐TOF/TOF MS

The protein band visible on the gel was cut out and added 200 μl of 30% acetonitrile solution containing 100 mmol/L ammonium bicarbonate. The mixture was shaken until colorless and dehydrated twice by adding 50 μl of anhydrous acetonitrile to obtain white micelles. Each tube was added 5 μl trypsin solution and left for 30–60 min at 4°C, so that the colloidal particles completely absorbed the enzyme solution. The excess enzyme solution was aspirated, and 20 μl of 25 mmol/L ammonium hydrogen carbonate solution was added to it, and the mixture was incubated at 37°C for 20 hr. The enzymatic hydrolysate was aspirated and transferred to a new centrifuge tube. The extraction solution was added to the original tube to ultrasonic extract. The enzymatic hydrolysate and the extract were rotary‐concentrated in vacuo and then re‐dissolved by adding 3 μl of TA60 solution (acetonitrile 600 μl, 10% trifluoroacetic acid (TFA) 10 μl, double‐distilled water 390 μl). A sample of 0.7 μl was aspirated, and then, 0.7 μl substrate was added. After drying, the sample was subjected to peptide mass fingerprint analysis using a mass spectrometer. The positive ion reflection mode and the automatic data acquisition mode were set for the primary mass spectrometry data acquisition. The scanning range was 700–3500 Da. Five to ten primary mass spectrometry peaks with good signal intensity were selected for secondary mass spectrometry analysis. Bio Tools software and Mascot software (http://www.matrixscience.com) were used, respectively, for integration and mass spectrometry data search in National Center for Biotechnology Information (NCBI).

#### 2.2.4 Amino acid analysis

##### Determination of free amino acids

The measurement method was determined according to Gao et al. (2011). Samples were filtered through micropore film (0.45 μm) before high‐performance liquid chromatography (HPLC) analysis. The relevant instrument and analysis parameters were as follows: TAG amino acid analysis column (3.9 mm i.d.×150 mm height), detect wavelength 254 nm, temperature 38°C, the solvent flow rate 1.0 ml⁄min, and the injection volume 10 μl. Solvent A of mobile phase comprised 60 ml of acetonitrile and 940 ml of 0.14 mol/L sodium acetate (pH 6.40, containing 0.05% triethylamine). Solvent B of mobile phase was 40% water and 60% acetonitrile by volume. A gradient elution procedure with solvent A and solvent B was carried out: 0 min, 5% B; 15 min, 20% B; 35 min, 40% B; 42 min, 65% B; 50 min, 80% B; 52 min, 5% B; and 60 min, 5% B, and run time was 60 min.

##### Determination of total nitrogen content

Concentrated hydrochloric acid was added into 5 ml Huangjiu samples, then hydrolyzed at 110°C for 22 hr, transferred to volumetric bottles, filtered, steamed and centrifuged, and determined according to Kjeldahl method by means of K2300 Kjeldahl Nitrometer (Gao et al., 2011).

##### Hydrophobicity analysis of amino acids

The hydrophobicity of amino acids was calculated according to Lozano's empirical formula, and the average hydrophobicity of proteins was further calculated according to the following formula (Lozano, Combes, & IBORRA, 1994).

$$H\varphi_{\mathrm{avg}}=\sum_{i=q}^{n}X_{i}\times H\varphi_{i}$$

*X_i_* represents the molar ratio of an amino acid; H*φ_i_* represents the hydrophobic value of an amino acid.

#### 2.2.5 Secondary structure analysis of haze protein by Fourier transform infrared spectroscopy (FTIR)

##### Sample pretreatment

After Huangjiu samples (500 ml) that have produced colloidal haze were laid aside for 5 days, the supernatant was separated siphoned, and then approximately, 80 ml of wine samples was left at the bottom. On the one hand, ammonium sulfate was added into the supernatant sample to 80% saturation. After overnight under 4°C, precipitate was collected by centrifugation, and the precipitate was transferred into the dialysis bag. After dialysis for 48 hr, the sample was freeze‐dried and analyzed by FTIR.

On the other hand, samples left at the bottom were shaken well and then centrifuged at 12,000 g for 30 min to collect the precipitate. The precipitate was packed into 1,000 Da dialysis bag and dialyzed in flowing water for 48 hr. After freeze‐drying, the precipitate was analyzed by FTIR.

##### FTIR analysis

Spectral processing was performed according to the method of Shevkani, Singh, Kaur, and Rana (2015). Infrared spectra were recorded using FTIR spectrometer. The spectrums were subjected to Fourier self‐deconvolution (FSD), second derivative (*SD*) analysis, and curve‐fitting procedures to locate peaks in amide I region using Peak Fit 4.12 software (Systat Software Company). The relative proportions of different secondary structures were determined by computing areas of spectral components (Gaussian peaks) assigned to a particular substructure in the amide I region.

## 3 RESULTS AND DISCUSSION

### 3.1 Two‐dimensional electrophoresis analysis of haze protein in Huangjiu

**FIGURE 1:**
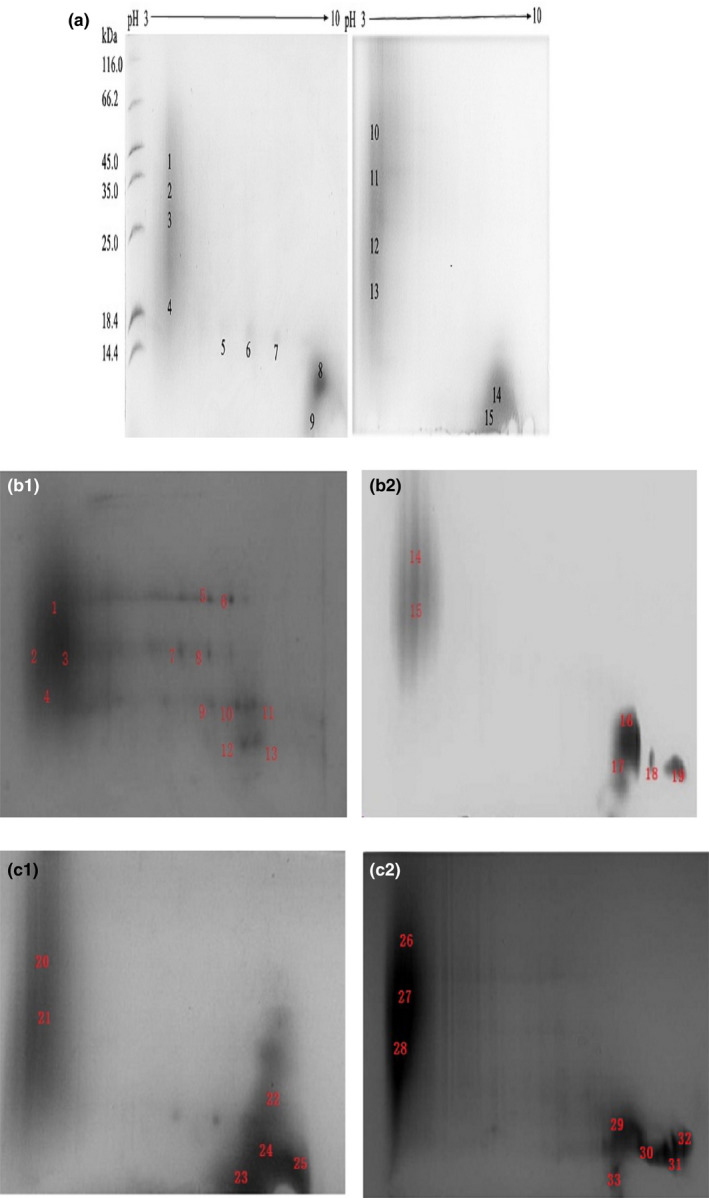
Two‐dimensional electrophoresis (2‐DE) profiles of haze protein in Huangjiu samples (7 cm, nonlinearity). (a) 5‐year old (point 1–9) and 10‐year old (point 10–15) Huangjiu in GuyueLongshan Shaoxing Wine Co. Ltd. (b) 5‐year old (point 1–13) and 10‐year old (point 14–19) Huangjiu from Tapai Shaoxing Wine Co. Ltd. (c) 5‐year old (point 20–25) and 10‐year old (point 26–33) Huangjiu from Kuaijishan Shaoxing wine Co. Ltd

The images of two‐dimensional electrophoresis (Figure 1) showed that the distribution of proteins in haze protein from different Huangjiu samples was similar, mainly including acidic proteins and low molecular weight alkaline terminal proteins. The protein spots distributing between acidic and alkaline terminals were fewer and light in color, which indicated that the content of these proteins in haze protein was fewer. Low molecular alkaline terminal protein spots were darker in color, suggesting a higher content of such proteins.

### 3.2 Identification of haze protein by MALDI‐TOF/TOF MS

The protein spots in Figure 1 were extracted for mass spectrometry analysis and retrieved in the NCBI database. A total of 48 spots were identified successfully and labeled in Figure 1. The information of each protein spot was shown in Table 1. A total of nine kinds of proteins were identified successfully in haze protein (Table 2). Among these proteins, there were six kinds of proteins derived from wheat, such as oat‐like protein b1, oat‐like protein, dimeric α‐amylase inhibitor, pathogenesis‐related protein (wheatwin l), pathogenesis‐related protein‐4, and chitinase II, which accounted for a large proportion and were the main sources of haze protein. The molecular weight of these proteins ranged between 13–16 kDa and 28–46 kDa. The proteins derived from rice were oat‐like protein and β‐amylase with a molecular weight of 33.4 kDa and 55.4 kDa, respectively. Considering the above, the haze protein was mainly derived from wheat and rice. The dimer alpha‐amylase inhibitor contributed the most significantly, and all were derived from wheat. The pathogen‐related protein (PR, PR‐4, chitinase II) contributed the most significant proportion after alpha‐amylase inhibitor, followed by oat‐like protein and beta‐amylase.

**TABLE 1:** Identification of protein spots corresponding to 2‐DE by MALDI TOF MS/MS

Location of sampling points	Identification results	Theoretical isoelectric point	Molecular weight/ (kDa)	Source of protein
a1	Oat‐like protein b1	8.08	33.8	Wheat
a2	Oat‐like protein	6.84	33.4	Rice
a3	Oat‐like protein	7.83	33.4	Wheat
a4	Dimeric alpha‐amylase inhibitor	5.58	15.1	Wheat
a5	Disease‐related protein (Wheatwin1)	5.8	13.9	Wheat
a6	Pathogenesis‐related protein−4	6.28	13.4	Wheat
a7	Pathogenesis‐related protein	6.97	14	Wheat
a8	Pathogenesis‐related protein−4	7	13.4	Wheat
a9	Dimeric alpha‐amylase inhibitor	8.03	13.5	Wheat
a10	Beta‐amylase	5.29	55.4	Rice
a11	Beta‐amylase 2	5.84	46.6	Rice
a12	Dimeric alpha‐amylase inhibitor	5.58	15.1	Wheat
a13	Dimeric alpha‐amylase inhibitor	6.86	13.3	Wheat
a14	Chitinase II	8.66	28.6	Wheat
a15	Dimeric alpha‐amylase inhibitor	8.03	13.5	Wheat
b1	Oat protein B	7.8	33.3	Wheat
b2	Dimeric alpha‐amylase inhibitor	5.8	15.6	Wheat
b3	Dimeric alpha‐amylase inhibitor	5.7	13.9	Wheat
b4	Dimeric alpha‐amylase inhibitor	5.8	15.6	Wheat
b5	Trypsin precursors	5.06	26.9	Wheat
b6	Oat protein A	8.42	19.2	Wheat
b7	Oat protein A	8.42	19.2	Wheat
b8	Dimeric alpha‐amylase inhibitor	5.8	15.7	Wheat
b9	Oat‐like protein precursor	8.42	20.7	Wheat
b10	Hypothetical protein OSJ_04535	10.36	29.7	Rice
b11	Hypothetical protein OSJ_04535	10.36	29.7	Rice
b12	Hypothetical protein OSJ_04535	10.36	29.7	Rice
b13	Hypothetical protein OSI_17439	8.95	36.3	Rice
b14	Dimeric alpha‐amylase inhibitor	5.8	15.6	Wheat
b15	Dimeric alpha‐amylase inhibitor	5.8	15.6	Wheat
b16	Oat‐like protein precursor	8.42	20.7	Wheat
b17	Oat‐like protein precursor	8.42	20.7	Wheat
b18	Protein H0313F03.18	8.88	36.1	Rice
b19	Hypothetical protein OSI_17439	8.95	36.3	Rice
c20	Dimeric alpha‐amylase inhibitor	5.8	15.7	Wheat
c21	Dimeric alpha‐amylase inhibitor	5.8	15.7	Wheat
c22	Oat‐like protein precursor	8.42	20.7	Wheat
c23	Hypothetical protein OSJ_04535	10.36	29.7	Rice
c24	Hypothetical protein OSJ_04535	10.36	29.7	Rice
c25	Hypothetical protein OSI_17439	8.95	36.3	Rice
c26	Dimeric alpha‐amylase inhibitor	5.8	15.7	Wheat
c27	Dimeric alpha‐amylase inhibitor	5.8	15.7	Wheat
c28	Dimeric alpha‐amylase inhibitor	5.7	13.9	Wheat
c29	Hypothetical protein OSJ_04535	10.36	29.7	Rice
c30	Oat‐like protein precursor	8.42	20.7	Wheat
c31	Hypothetical protein OSI_17439	8.95	36.3	Rice
c32	Hypothetical protein OSI_17439	8.95	36.3	Rice
c33	Oat‐like protein precursor	8.42	20.7	Wheat

**TABLE 2:** Identified haze proteins in Huangjiu by MALDI‐TOF/TOF MS

Protein name	Login number	Theoretical isoelectric point	Relative molecular weight/kDa	Species origin
Avenin‐like b1	gi∣122232330	8.08	33.788	Wheat
Avenin‐like protein	gi∣156630234	6.84	33.425	Rice
Avenin‐like protein	gi∣363981,043	7.83	33.401	Wheat
Dimeric alpha‐amylase inhibitor	gi∣65993781	5.58	15.118	Wheat
Pathogenesis‐related protein−4, Wheatwin1	gi∣453118	5.80	13.929	Wheat
Pathogenesis‐related protein−4 (PR−4)	gi∣6048567	6.28	13.430	Wheat
Pathogenesis‐related protein	gi∣1588926	6.97	14.013	Wheat
Pathogenesis‐related protein−4 (PR−4)	gi∣6048596	7.00	13.442	Wheat
Dimeric alpha‐amylase inhibitor	gi∣54778505	8.03	13.500	Wheat
Beta‐amylase	gi∣169777	5.29	55.400	Rice
Beta‐amylase 2	gi∣482677643	5.84	46.586	slender false brome grass
Dimeric alpha‐amylase inhibitor	gi∣65993781	5.58	15.118	Wheat
Dimeric alpha‐amylase inhibitor	gi∣66841026	6.86	13.340	Wheat
Class II chitinase	gi∣62465514	8.66	28.599	Wheat
Dimeric alpha‐amylase inhibitor	gi∣54778505	8.03	13.500	Wheat

It was previously reported that dimer alpha‐amylase inhibitor and oat protein were also the components of beer haze protein (Joshi, Panesar, Rana, & Kaur, 2017). Alpha‐amylase inhibitor was first discovered in wheat seeds and was found to be sugar hydrolase inhibitor, which had a strong inhibitory effect on alpha‐amylase from different sources (Silano et al., 1975; Svensson, Fukuda, Nielsen, & Bønsager, 2004). Alpha‐amylase inhibitors can inhibit the activity of endogenous amylase, so that the starch ingested cannot be hydrolyzed, blocking the main source of energy, and thus can protect plants against insect damage (Iulek et al., 2000; Silano et al., 1975). Alpha‐amylase inhibitors have good acid–base tolerance since stable protein properties and structure in the range of pH 4–11 (Johnson et al., 2018). After action for 30 min at 70°C there was almost no change in its activity, and the activity under 80°C for 10 min was only reduced by about 10%. Based on this, we further deduced that the reason why Huangjiu showed better thermal stability during the process of decocting (heat sterilization) was likely that only a small part of the alpha‐amylase inhibitors was inactivated, denatured, or coagulated, and most of them still maintained in Huangjiu. After a long period of storage, the alpha‐amylase inhibitor gradually denatured and solidified to form turbidity.

The pathogenesis‐related proteins (PRs) are general water‐soluble proteins induced by pathogens in plants. The main function of PR protein is to participate in the plants’ disease resistance reaction, degrade the pathogen toxins, and inhibit the binding of the viral oat protein and plant molecules, and form a plant defense system (Haque et al., 2014; Naz, Bano, Wilson, Guest, & Roberts, 2014; Wang et al., 2010). PRs are a class of monomeric proteins with a relative molecular weight of 10–40 kDa, and most of the PRs can tolerate low pH, heavy metal, protease, and high temperature (Gamir et al., 2017).

According to their amino acid sequence similarity, PRs can be divided into 14 families of PR‐1–14. Among them, chitinase II belongs to the PR‐3 family, which can degrade the cell wall of pathogenic bacteria and improve the disease resistance of plants. Chitinase was also found in wine turbidity (Iimure & Sato, 2013). Therefore, it was possible that because of the PR stability, PRs could not be removed even after being decocted and filtered during the brewing process. As a result, PRs eventually were retained in the finished wine and were able to contribute to perceived turbidity in the Huangjiu.

Oat‐like protein mainly found in wheat endosperm is a low molecular weight gluten. It is divided into subtypes A and B (Cao et al., 2018). Of the 168 amino acids in the subtype A protein, 36 were glutamic acid units, accounting for 21% of total amino acid. In the B‐type protein, on the other hand, 80 of the 284 amino acids were glutamic acid, amounting to 28% of the total. This was consistent with a higher proportion of glutamic acid content in the following amino acid analysis of haze protein (Table 3). In addition, oat‐like proteins containing highly redundant cysteine residues and disulfide bonds within and between molecules can form polymers, which have an important impact on the formation of turbidity (Hosseini, Kadivar, & Shahedi, 2012; Mirmoghtadaie, Kadivar, & Shahedi, 2009).

**TABLE 3:** Amino acid compositions of haze protein and supernatant protein in Huangjiu

Amino acid	Hydrophobicity	Haze protein/%	Supernatant protein/%
Asp	0	8.54	8.67
Glu	0	20.48	31.98
Ser	−0.3	4.73	7.53
His	0.5	2.20	3.05
Gly	0	6.06	6.17
Thr	0.4	4.05	3.89
Arg	0.75	7.59	3.94
Ala	0.5	6.07	4.26
Try	2.3	3.56	1.44
Cys	1	3.81	1.11
Val	1.59	5.10	5.25
Met	1.07	1.76	7.64
Phe	2.5	3.51	3.57
Ile	2.95	3.06	3.84
Leu	1.8	7.00	4.86
Lys	1.5	2.34	2.48
Pro	2.6	10.12	7.31
Hφavg （Average hydrophobicity of amino acids）	/	0.92	0.79

### 3.3 Amino acid analysis of haze protein

The amino acid components of haze protein and supernatant protein in Huangjiu were analyzed, and the results were displayed in Table 3. The hydrophobic value indicated the hydrophobicity of the amino acid, and the larger the value, the stronger the hydrophobicity. In the haze protein, glutamic acid content was the highest, accounting for 20.48% of the total amino acid content, followed by proline and aspartic acid, accounting for 10.12% and 8.54% of the total amino acid content, respectively. Among the supernatant protein, the highest content was also glutamic acid, accounting for 31.98% of the total amino acid content, followed by aspartic acid and serine, accounting for 8.67% and 7.53%, respectively. The hydrophobic value of hydrophilic glutamic acid and aspartic acid was equal to zero. And the hydrophobic value of serine was −0.3, which was the most hydrophilic amino acid. The content of serine in the supernatant protein was 7.53%, which was 1.59 times content of the haze protein. The hydrophobic value of proline was 2.6, the relatively larger hydrophobic value of amino acids. The proportion of proline in the haze protein was 1.38 times that of the supernatant protein. The content of the hydrophobic amino acids in the haze protein was 60.19%, and the content in the supernatant protein was 45.65%, and the former was 1.32 times the latter. The average hydrophobic value of protein amino acids in the supernatant was 0.79, and the average hydrophobic value of the haze protein amino acids was 0.92. The latter was 16.46% higher than the former. It indicated that the haze protein was more hydrophobic.

### 3.4 Secondary structure analysis of haze protein

**FIGURE 2:**
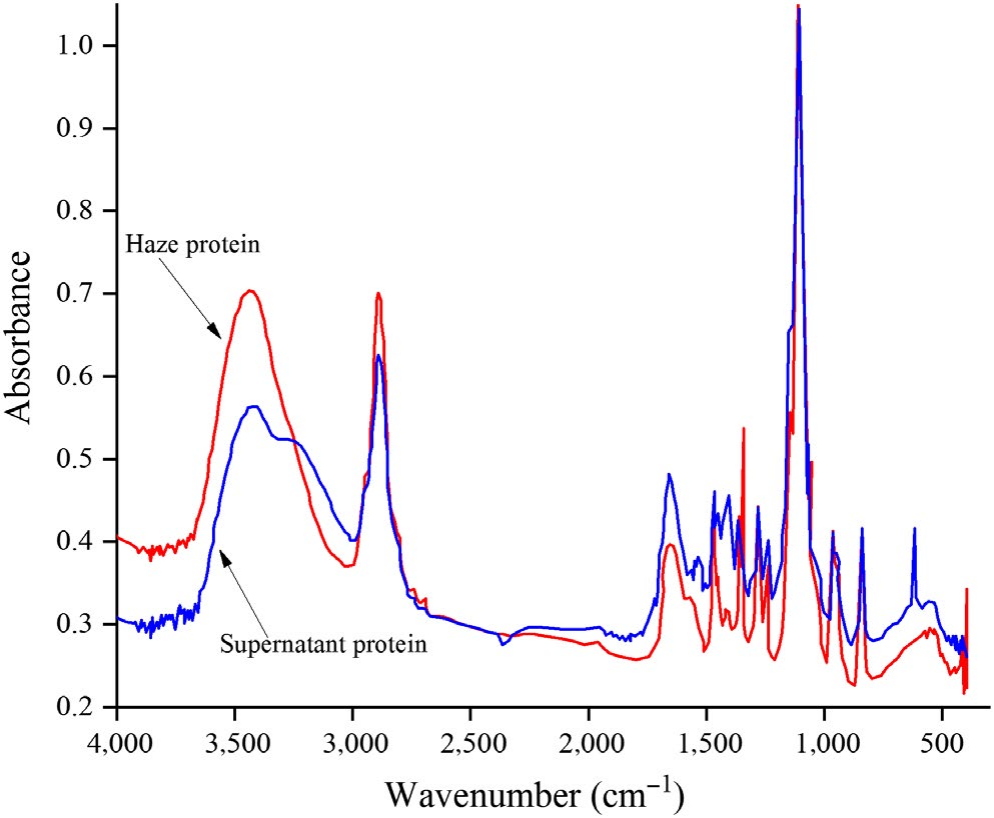
Full band Fourier transform infrared spectrogram of haze protein and supernatant protein

**FIGURE 3:**
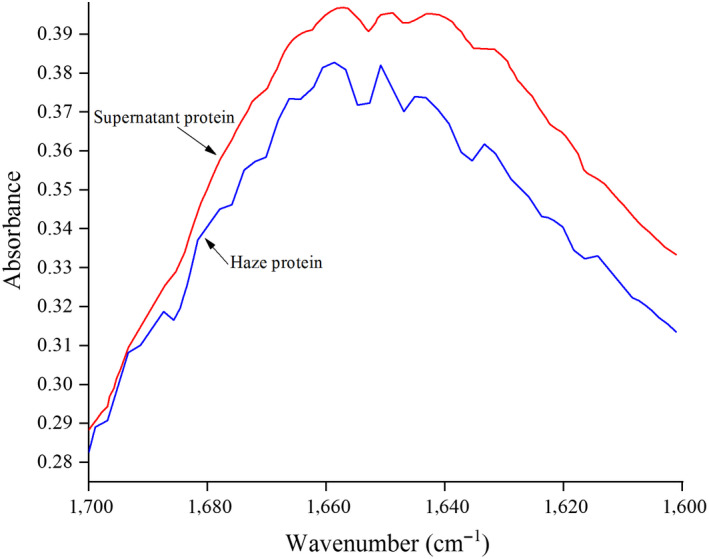
Amide I band Fourier transform infrared spectrogram for haze protein and supernatant protein

Regardless of the conformation of the side chains and the spatial arrangement of the entire peptide chain, the secondary structure of a protein refers to the spatial structure of the polypeptide chains. Common secondary structures include α‐helix, β‐sheet, β‐turn, and random curl (Zhao, Chen, Xue, & Lee, 2008). FTIR, an effective way to study the relationship between protein conformation and function, has been widely used in the study of protein secondary structure. As displayed in Figure 2, the absorbance of the haze protein was significantly higher than the supernatant protein in the band of 4000‐3200/cm, while in the amide I band (1700‐1600/cm), the absorbance of the supernatant protein was greater than the haze protein (Figure 3). So, it was suggested that there were differences in protein structure between haze and supernatant protein.

**FIGURE 4:**
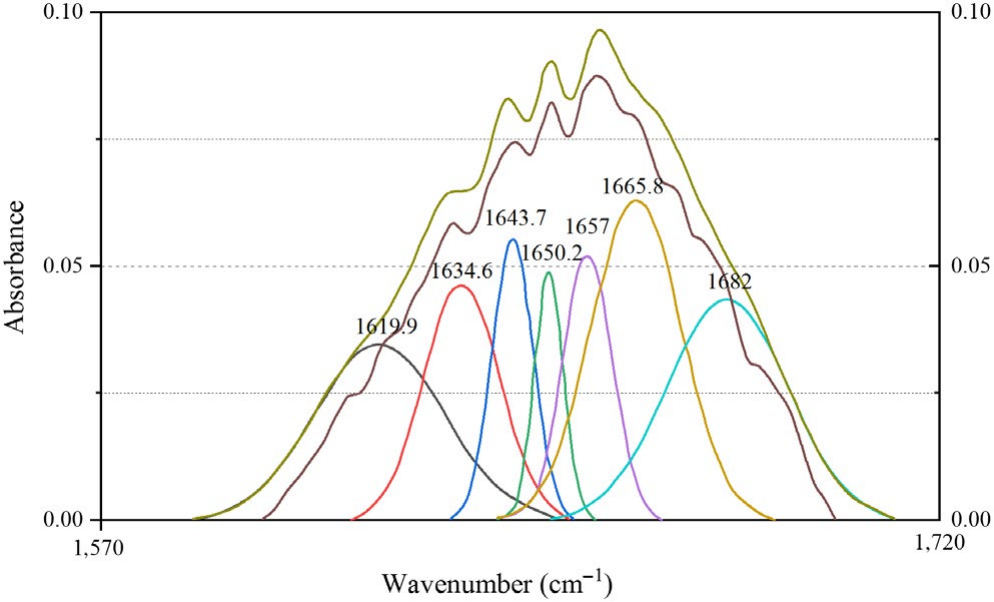
Curve‐fitted map of secondary structure of haze protein in amide I band

**FIGURE 5:**
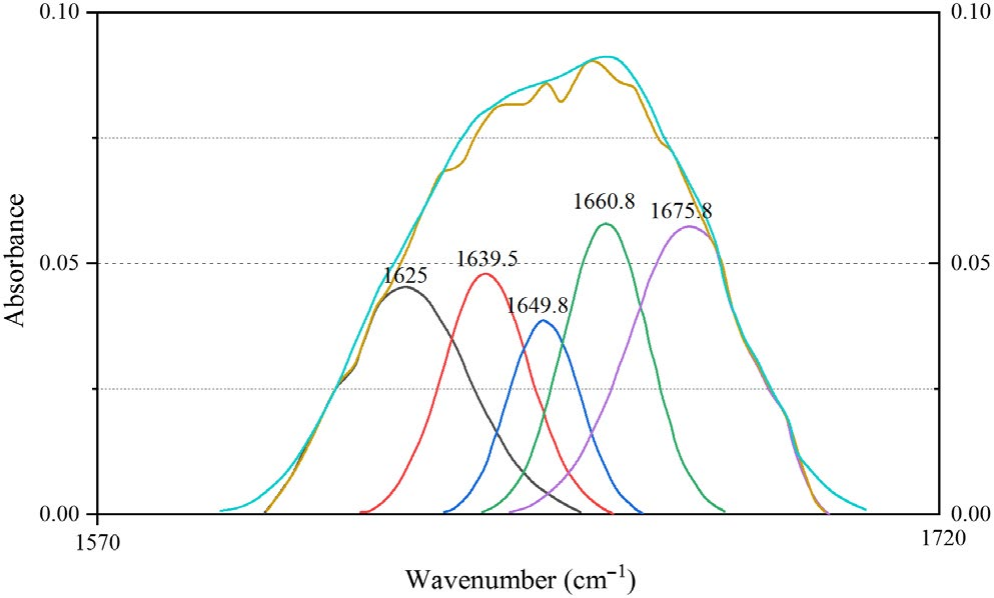
Curve‐fitted map of secondary structure of supernatant protein in amide I band

The absorbance data of amide I band in the spectrogram were intercepted, and the spectrogram was fitted by Peak Fit 4.12 software (Systat Software Company). An obvious difference between the haze and supernatant protein was observed in the amide I band fit map (Figure 4, Figure 5). Seven distinct peaks were obtained in haze protein, while there were only five peaks in the supernatant protein. The secondary structure corresponding to each peak and the relative percentages of the secondary structures in the haze and supernatant protein were compared in Tables 4 and 5, respectively. Consistent with Figures 4 and 5, an obvious difference between the haze and supernatant protein in the secondary structure was revealed. For example, the supernatant protein did not contain α‐helix structure, while the α‐helix content in the haze protein was 15.91%. Moreover, the content of β‐sheet, random curl, and β‐turn in the supernatant protein was 26.9%, 25.3%, and 2.4% higher than that of the haze protein, respectively. It has been reported that protein secondary structure was related to surface hydrophobicity (Van Dijk, Hoogeveen, & Abeln, 2015). In addition, the water solubility was negatively correlated with the proportion of α‐helix content and positively correlated with the proportion of β‐sheet and random curl, but no correlation with the proportion of β‐turn content (Gao et al., 2011; Qu et al., 2018; Wang et al., 2011). Considering the above results and discussion, the main structural features of haze protein in Huangjiu were the high content ratio of α‐helix, the low content ratio of β‐sheet and random curl.

**TABLE 4:** Secondary structure analysis of haze protein and supernatant protein during amide I band by FTIR

Peak number	Supernatant protein			Haze protein		
	Position	Peak area	Corresponding structure	Position	Peak area	Corresponding structure
1	1625.0	23.56	Beta folding	1619.9	17.90	Beta folding
2	1639.5	16.87	Beta folding	1634.6	13.96	Beta folding
3	1649.8	11.26	Irregular curl	1642.7	8.99	Irregular curl
4	1,660.8	19.81	Beta angle	1,650.2	5.61	Alpha helix
5	1675.8	28.48	Beta angle	1657.0	10.30	Alpha helix
6				1665.8	22.53	Beta angle
7				1682.0	20.71	Beta angle

**TABLE 5:** Percentage of secondary structure compositions of proteins in the haze and the supernatant (%)

Structure	Supernatant proteins	Haze proteins
Beta folding	40.43	31.86
Irregular curl	11.26	8.99
Alpha helix	0	15.91
Beta angle	44.29	43.24

In the production of Huangjiu, the sterilization was usually carried out under the conditions of 80–85°C for about 40 min in order to ensure the biological stability of bottled Huangjiu (Yang et al., 2019). It was possible that high‐temperature sterilization destroyed the high‐grade structure (including the secondary structure) of the protein, resulting in enhanced hydrophobicity of the protein, reduced water solubility, and eventually turbidity.

## 4 CONCLUSIONS

The main composition and source of colloidal haze protein in Shaoxing Huangjiu were analyzed. Six kinds of proteins derived from wheat and two kinds of rice proteins were identified using MALDI‐TOF/TOF MS in the haze protein, including oat‐like protein b1, oat‐like protein, dimeric alpha‐amylase inhibitor, pathogenesis‐related protein, pathogenesis‐related protein‐4, chitinase II, and beta‐amylases. The molecular weight of the haze protein in Huangjiu mainly ranged between 13–16 kDa and 28–55.4 kDa. Amino acid analysis of haze protein revealed that the content of glutamic acid was the highest in haze protein, followed by proline and aspartic acid. Moreover, the average hydrophobic value of haze protein amino acids was 0.92, which was 16.46% higher than that of supernatant protein amino acids in Huangjiu. Besides, FTIR analysis showed that the content of α‐helix structure in haze protein was 15.91%, while the supernatant protein did not contain α‐helix. However, the contents of β‐fold and random curl structure in haze protein were significantly lower than that in supernatant protein. It indicated that the high hydrophobicity and low water solubility of amino acid compositions were the main characteristics of the proteins which formed the colloidal haze in Huangjiu.

## CONFLICT OF INTEREST

The authors declare that they do not have any conflict of interest.

## ETHICAL REVIEW

This study does not involve any human or animal testing.

## INFORMED CONSENT

Written informed consent was obtained from all study participants.
